# Clinical characteristics and prognosis of cystic degeneration in retroperitoneal schwannoma: A retrospective study of 79 patients

**DOI:** 10.1002/cam4.5411

**Published:** 2022-11-28

**Authors:** Jianchun Xiao, Lizhu Cai, Jun Pu, Wei Liu, Congwei Jia, Xiaodong He

**Affiliations:** ^1^ Department of General Surgery Peking Union Medical College Hospital, Chinese Academy of Medical Sciences Beijing China; ^2^ Institute of Clinical Medicine Chinese Academy of Medical Sciences & Peking Union Medical College Beijing China

**Keywords:** malignant, retroperitoneal schwannoma clinical characteristics, cystic degeneration, prognosis, retroperitoneal schwannoma

## Abstract

**Background and Purpose:**

Diagnosis of retroperitoneal schwannoma (RS), especially cystic RS, is frequently missed or delayed owing to its rarity, location, nonspecific symptoms, and similarities with other tumors on various imaging modalities. This study aimed to determine associations between clinical, radiological, and histopathologic features and outcome.

**Materials and Methods:**

Seventy‐nine patients with pathologically confirmed RS who underwent tumor resection between June 2010 and June 2020 were retrospectively reviewed and analyzed. Patients were stratified into three groups according to degree of tumoral cystic degeneration.

**Results:**

Cystic degeneration was significantly associated with multiple foci (*p* = 0.025), calcification (*p* = 0.012), and hemorrhage (*p* = 0.000), but not size (*p* = 0.08), high Ki‐67 (*p* = 0.094), malignancy (*p* = 0.115; prevalence of cystic degeneration in the benign and malignant groups were 53.9% vs 100%), rough margin (*p* = 0.162), or irregular shape (*p* = 0.369). Malignant RS was significantly associated with multiple lymph nodes enlargement (*p* = 0.034). Tumor size, margins, shape, or/and multiplicity did not significantly differ between benign and malignant tumors. No recurrence occurred in patients with benign RS (mean follow‐up, 45 months). All malignant tumors recurred; mean time to recurrence was 11.4 months (mean follow‐up, 33 months).

**Conclusion:**

Since RS is misdiagnosed mostly as malignancy and diagnosis is often delayed, a suspicion is necessary for diagnosis when atypical features are present. In RS, cystic degeneration was not associated with tumor size, Ki‐67, or malignancy; however, it was significantly associated with multiple foci, calcification, and hemorrhage. Cystic degeneration and related factors are useful for the diagnosis of RS. Malignant RS should be considered when a mass involves multiple lymph nodes. Margins, morphology, and size are not associated with malignancy. Pathological tumor type, tumor location, and adjacent anatomic structures are associated with outcome.

## Graphical abstract

Retrospective study of 79 patients revealed a high prevalence of cystic degeneration in retroperitoneal schwannoma (RS). Related factors of cystic degeneration and malignancy were explored. We also presented clinical features, image and histopathologic characteristics of RS.

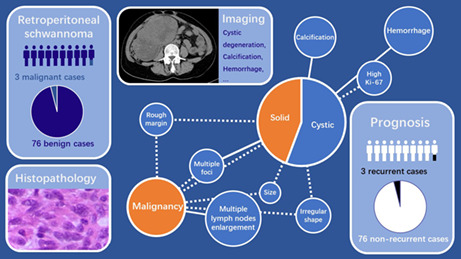

## 1 BACKGROUND

Schwannoma,^1^ also known as neurilemmoma or neurinoma, is the most common benign neurogenic tumor.^2^ It is a well‐encapsulated spindle cell tumor that originates from Schwann cells of the peripheral nervous system.^3^ Most are solitary, localized, and grow slowly; they typically present as a painless soft‐tissue mass and cause few nonspecific symptoms.^4^ Although schwannomas may occur at any age, peak incidence is in the third through fifth decades of life. Both genders are affected equally.^1^, ^3^ The overwhelming majority occur along nerves anywhere throughout the whole body,^5^, ^6^ with a predilection for the head, neck, and flexor surfaces of the extremities.^7^ Other commonly affected areas include the paravertebral region, posterior mediastinum, retroperitoneum, juxtarenal area, and pelvis.^4^

Retroperitoneal schwannoma (RS) is located in the retroperitoneum, which is posterior to the parietal peritoneum and extends inferiorly from the diaphragm to the pelvis, where communication with the peritoneal cavity and extraperitoneal pelvic space is possible.^8^ The retroperitoneal space is bounded posteriorly by the quadratus lumborum muscles and is connected with the extraperitoneal fat space. Therefore, RSs can grow into the posterior mediastinum, lateral abdominal wall, and extraperitoneal pelvic space. As a result, RSs may be considerably larger than those at other sites. Symptoms are usually related to mass effect on adjacent structures. However, neurological symptoms and pain are unusual because the retroperitoneum is pliable. Preoperative diagnosis of RS is challenging because they are rare, cause nonspecific symptoms, and appear similar to other retroperitoneal tumors on imaging studies. In particular, diagnosis of cystic RS is frequently missed or delayed, as most radiologists and clinicians are not familiar with it.

Ancient schwannoma^9^ is described as a long‐standing schwannoma with signs of degenerative changes, including cyst formation, calcification, hemorrhage, and hyalinization. In particular, retroperitoneal totally cystic schwannoma is relatively rare, while most radiologists and clinicians lack awareness of it. Most schwannomas are solid and enhanced during the arterial phase on contrast‐enhanced CT, and diagnosis is comparatively easy. However, morphologically changed RS, such as cystic RS, is sometimes difficult to differentiate from other cystic lesions. The rare degenerative histological changes frequently pose diagnostic difficulty by mimicking benign lesions^5^ or malignancies.^10^ The challenge is how to preoperatively diagnose and determine the prognosis of cystic RS.

Malignant schwannoma, also known as malignant peripheral nerve sheath tumor (MPNST), is a high‐grade soft‐tissue sarcoma with high rate of local recurrence and metastasis and relatively poor prognosis.^11^ Local recurrence is the most frequent complication in MPNST after surgical resection, mainly due to positive resection margins and a high malignancy grade.^2^

Treatment and prognosis differ between RS, MPNST, and other malignant retroperitoneal tumors. Benign schwannoma requires only complete lesion resection,^9^, ^12^ while malignant tumors require resection combined with postoperative radiotherapy and/or chemotherapy. Accurate preoperative diagnosis assists selection of appropriate treatment and enables avoidance of unnecessary extensive surgeries, such as adrenalectomy,^13^, ^14^ pancreatectomy,^15^ and regional lymph node dissection.

Understanding the clinical behavior, radiologic findings, and pathological characteristics of RS is imperative to provide a correct preoperative diagnosis. In this study, we reviewed clinical characteristics, radiological findings, treatment, and outcomes in 79 RS patients who underwent surgical resection. Our aim was to determine associations between clinical, radiological, and histopathologic features and outcome and provide clinical data that will assist fellow clinicians in diagnosing RS.

## 2 MATERIALS AND METHODS

This retrospective study evaluated 79 Chinese patients who were treated successfully by complete surgical resection, pathologically diagnosed with RS and admitted to Peking Union Medical College Hospital (PUMCH), Beijing, China, between June 2010 and June 2020.

This study collected demographic characteristics (sex and age), clinical data (manifestations, laboratory findings, and radiological findings), therapeutic regimen, pathological manifestations, and follow‐up data (complications and recurrence) by reviewing the medical records of various patients included.

In all subjects, the diagnosis of RS was confirmed by pathological examination at PUMCH. All patients underwent both imaging and clinical examinations. The imaging findings included tumor location, shape, size, amount, margin, signal intensity, internal mass enhancement pattern, blood flow information, secondary degeneration (cyst formation, hemorrhage, and calcification), and regional lymph nodes. A joint consultation was implemented if a controversial conclusion needed to be resolved. The pathological and laboratory records included the tumor size, gross macroscopic findings, HE staining, and immunohistochemical results for surgical resection specimens, which were marked by S‐100, epithelial membrane antigen (EMA), smooth muscle actin (SMA), glial fibrillary acidic protein (GFAP), and Ki‐67. Our cohort was obtained from the histologic database of PUMCH.

Laboratory studies, including plasma and urine catecholamine studies, of schwannomas showed normal levels of hormone secretion in all patients.

All statistical analyses were performed with IBM SPSS (version 22.0; IBM Corp.). Categorical variables are summarized as numbers (percentages) and were analyzed using the chi‐square test or Fisher's test. Continuous variables are expressed as the mean and standard deviation, and Student's *t*‐test was used for comparisons between the two subtypes. *p* < 0.05 was considered significant.

## 3 RESULTS

Forty‐four tumors (55.7%) exhibited heterogeneous cyst formation. Patients were stratified into three groups according to degree of tumoral cystic degeneration: group 1, area of cystic degeneration >50% of the entire tumor area; group 2, area of cystic degeneration <50% of the entire tumor area; and group 3, no cystic degeneration (solid group). The cystic degeneration group comprised groups 1 and 2.

### 3.1 Patient demographics

Patient characteristics are summarized in Table 1. Median age was 50 years (range, 15–78; IQR, 22) . Thirty‐two patients were men and 47 were women. Mean age was higher in group 1 than group 3. The male/female ratio did not significantly differ between the groups. Notably, RSs occur at all ages, with a peak incidence between the fourth and sixth decades. In this study, all patients were adults except for one. Subgroups aged 20–44 years and 45–64 years had a higher prevalence than those aged 15–19 years and more than 65 years. Group 1, without a sex predilection, on average, was older than group 3. Group 3, showed a sex predilection (females), had a difference close to statistical significance (*p* = 0.075). Depending on the age group, the cystic degeneration rates for RS were higher among people between 45 and 64 years of age than those between 20 and 44 years of age. The mean age at which cystic degeneration occurred was 51.49 years, and these patients tended to be older than those without cystic degeneration or with low cystic degeneration, although the difference was not significant (*p* = 0.178 and *p* = 0.175). The occurrence of RS without cystic degeneration was prominent among young adults.

**TABLE 1:** Demographic and clinical characteristics of 79 patients

	Group 1(*n*)	Group 2 (*n*)	Group 3 (*n*)	Total (*n*)	*p* value	Cystic degeneration group (*n*)	Solid group (*n*)	*p* value
Number of patients	17 (21.5%)	27 (34.2%)	35 (44.3%)	79 (100%)		44 (55.7%)	35 (44.3%)	
Age (years)								
Range of ages at surgery	27 ~ 74	15 ~ 78	23 ~ 72	15 ~ 78		15 ~ 78	23 ~ 72	
Median age	50	51	45	50		50.5	45	
IQR	25	26	23	21		20.75	23	
Mean age	51.94 (S.D. = 13.9)	45.67 (S.D. = 15.1)	46.00 (S.D. = 15.96)	47.17 (S.D. = 14.81)	0.327	48.09 (S.D. = 14.80)	46.00 (S.D. = 15.96)	0.537
Age group					0.460			0.527
Age ≤ 20	0	1 (3.7%)	0	1 (1.3%)		1 (2.3%)	0	
20 < Age ≤ 44	5 (29.4%)	11 (40.7%)	17 (48.6%)	33 (41.8%)		16 (36.4%)	17 (48.6%)	
44 < Age ≤ 64	9 (52.9%)	14 (51.9%)	14 (40%)	37 (46.9%)		23 (52.3%)	14 (40%)	
Age ≥ 65	3 (17.6%)	1 (3.7%)	4 (11.4%)	8 (10.1%)		4 (9.1%)	4 (11.4%)	
Sex					0.239			0.075
Male	9 (52.9%)	13 (48.1)	11 (31.4%)	33 (41.8%)		22 (50%)	11 (31.4%)	
Female	8 (47.1%)	14 (51.9%)	24 (68.6%)	46 (58.2%)		22 (50%)	24 (68.6%)	
Clinical manifestations								
Incidental finding during a routine health examination	13 (76.5%)	23 (85.2%)	33 (94.3%)	69 (87.3%)		36 (81.8%)	33 (94.3%)	
Asymptomatic mass	6 (35.3%)	17 (63%)	27 (77.1%)	50 (63.3%)		23 (52.3%)	27 (77.1%)	
Abdominal pain	5 (29.4%)	4 (14.8%)	2 (5.7%)	12 (15.2%)		9 (20.5%)	2 (5.7%)	
Waist and back pain	4 (23.5%)	4 (14.8%)	3 (8.6%)	11 (13.9%)		8 (18.2%)	3 (8.6%)	
Palpable abdominal mass by self‐check	1 (5.9%)	4 (14.8%)	2 (5.7%)	7 (8.7%)		5 (11.4%)	2 (5.7%)	
Mild abdominal distension	2 (11.8%)	2 (7.4%)	2 (5.7%)	6 (7.6%)		4 (9.1%)	2 (5.7%)	
Nausea and vomiting	1 (5.9%)	2 (7.4%)	0	3 (3.8%)		3 (6.8%)	0	
Nerve compression	1 (5.9%)	1 (3.7%)	0	2 (2.5%)		2 (4.5%)	0	
Medical history								
Cesarean section	1 (5.9%)	2 (7.4%)	2 (5.7%)	5 (6.3%)		3 (6.8%)	2 (5.7%)	
Cesarean section plus renal cystectomy	0	1 (3.7%)	0	1 (1.3%)		1 (2.3%)	0	
Appendectomy	0	1 (3.7%)	0	1 (1.3%)		1 (2.3%)	0	
Hysterectomy	0	1 (3.7%)	1 (2.9%)	2 (2.5%)		1 (2.3%)	1 (2.9%)	
Cholecystectomy	0	0	2 (5.7%)	2 (2.5%)		0	2 (5.7%)	
Physical examination								
Palpable abdominal mass	4 (23.5%)	5 (18.5%)	4 (11.4%)	13 (16.5%)	0.510	9 (20.5%)	4 (11.4%)	0.222
Tenderness	2 (11.8%)	0	2 (5.7%)	4 (5.1%)	0.217	2 (4.5%)	2 (5.7%)	0.601
Percussion pain	1 (5.9%)	1 (3.7%)	1 (2.9%)	3 (3.8%)	0.866	2 (4.5%)	1 (2.9%)	0.586
Decreased sensation	0	1 (3.7%)	0	1 (1.3%)	0.158	1 (2.3%)	0	0.369
Mean diameter of the palpable abdominal mass (cm)	12.27 (S.D. = 7.31)	11.34 (S.D. = 4.87)	4.8 (S.D. = 1.73)	9.39 (S.D. = 5.55)	0.118	11.69 (S.D. = 5.39)	4.8 (S.D. = 1.73)	0.035

The medical and family histories of the patients were unremarkable. None of the 79 patients had prior radiotherapy. Five patients had undergone cesarean section, one patient had undergone appendectomy, two patients had undergone hysterectomy, and two patients had undergone cholecystectomy. One patient had undergone cesarean section plus renal cystectomy, but the remainder had not undergone surgery. The variables sex, age, and medical history did not present a significance difference between the three groups.

### 3.2 Clinical manifestations

Most RSs were incidentally discovered by imaging modalities such as ultrasonography, x‐ray, CT, and magnetic resonance imaging (MRI). Asymptomatic abdominal mass was the most frequent clinical presentation (63.3%). Atypical symptoms resulting from organ displacement^16^ included mild/dull abdominal pain (*n* = 12, 15.2%), vague waist and back pain (*n* = 11, 13.9%), abdominal mass (*n* = 7, 8.7%), mild abdominal distension and discomfort (*n* = 6, 7.6%), nausea and vomiting (*n* = 3, 3.8%), and mild numbness and pain owing to nerve compression (*n* = 2, 2.5%). Symptom duration varied from 1 week to 10 years.

Physical examinations revealed that the most common abnormal findings noted among RS patients were abdominal mass (*n* = 13, 16.3%), tenderness (*n* = 5, 6.3%), and percussion pain (*n* = 3, 3.8%). All three patients with percussion pain complained of pain located in the paravertebral region. Tenderness, which was observed in 4 patients, was located in the pelvic cavity or fossa iliaca. Thirteen patients had a palpable abdominal mass in the following locations: pelvic cavity (*n* = 5), fossa iliaca (*n* = 4), paravertebral region (*n* = 2), periaortic region (*n* = 1), and pancreatic region (*n* = 1). Palpable abdominal masses ranging from 3.1 to 20.3 cm in diameter were hard, with well‐defined borders and smooth surfaces. The mean diameter of the palpable abdominal masses with mild tenderness in three patients was no larger than that of those in patients with only one sign (10.1 cm vs 7.3 cm, *p* = 0.47). The mean diameter of the palpable abdominal masses in the cystic degeneration group (groups 1 and 2) was significantly larger than that of those in the solid group (group 3; *p* = 0.035).

### 3.3 Imaging manifestations

The imaging findings are shown in Table 2. Abdominal ultrasonography, plain CT, contrast‐enhanced CT, and MRI were performed in 35, 76, 60, and 25 patients, respectively.

**TABLE 2:** Imaging findings in 79 patients with RS

Patients	Group 1 (*n*)	Group 2 (*n*)	Group 3 (*n*)	Total (*n*)	*p* value	Cystic degeneration group (*n*)	Solid group (*n*)	*p* value
Side					0.375			0.634
Left	8 (47.1%)	12 (44.4%)	19 (54.3%)	39 (49.4%)		20 (45.5%)	19 (55.9%)	
Right	6 (35.5%)	14 (51.9%)	13 (37.1%)	33 (41.8%)		20 (45.5%)	13 (38.2%)	
Intermediate	3 (17.6%)	1 (3.7%)	2 (5.7%)	6 (7.6%)		4 (9.1%)	2 (5.9%)	
Diameter								
Range (cm)	3.7 ~ 20.3	2.1 ~ 16.3	2.2 ~ 14.1	2.1 ~ 20.3		2.1 ~ 20.3	2.2 ~ 14.1	
<5 cm	6 (37.5%)	11 (40.7%)	18 (52.9%)	35 (45.5%)		17 (39.5%)	18 (%)	
5–10 cm	8 (50.0%)	12 (44.4%)	13 (38.2%)	33 (42.9%)		20 (46.5%)	13 (%)	
10–15 cm	1 (6.3%)	1 (3.7%)	3 (8.8%)	5 (6.5%)		2 (4.7%)	3 (%)	
15–20 cm	0	3 (11.1%)	0	3 (3.9%)		3 (7.0%)	0	
≥20 cm	1 (6.3%)	0	0	1 (1.3%)		1 (2.3%)	0	
Mean diameter (cm)	6.8 0 (S.D. = 4.13)	6.87 (S.D. = 3.99)	5.44 (S.D. = 2.74)	6.23 (S.D. = 3.54)	0.22	6.85 (S.D. = 3.99)	5.44 (S.D. = 2.74)	0.08
Median diameter (cm)	6.0	5.4	4.3	5.1		6.0	4.3	
IQR (cm)	5.3	4.0	2.3	2.2		3.6	2.3	
Location					0.017			0.010
Paravertebral region with spinal canal extension	1 (5.9%)	1 (3.7%)	4 (11.8%)	6 (7.6%)		2 (4.5%)	4 (11.8%)	
Paravertebral region without spinal canal extension	4 (23.5%)	10 (37.0%)	2 (5.9%)	16 (20.3%)		14 (31.8%)	2 (5.9%)	
Pancreatic region	3 (17.6%)	1 (3.7%)	1 (2.9%)	5 (6.3%)		4 (9.1%)	1 (2.9%)	
Adrenal region	1 (5.9%)	2 (7.4%)	9 (26.5%)	12 (15.2%)		3 (6.8%)	9 (26.5%)	
Close to the renal hilus	0	7 (25.9%)	12 (35.3%)	19 (24.1%)		7 (15.9%)	12 (35.3%)	
Periaortic region	3 (17.6%)	3 (11.1%)	2 (5.9%)	8 (10.1%)		6 (13.6%)	2 (5.9%)	
Pelvic cavity	3 (17.6%)	1 (3.7%)	3 (8.8%)	7 (8.9%)		4 (9.1%)	3 (8.8%)	
Fossa iliaca	2 (11.8%)	2 (7.4%)	1 (2.9%)	5 (6.3%)		4 (9.1%)	1 (2.9%)	
Total	17	27	34	78		34	34	
Amount of foci					0.071			0.025
Single foci	15 (88.2%)	23 (85.2%)	35 (100%)	73 (92.4%)		38 (86.4%)	35 (100%)	
Multiple foci	2 (11.8%)	4 (14.8%)	0	6 (7.6%)		6 (13.6%)	0	
Morphology					0.111			0.369
Rounded	14 (82.4%)	26 (100%)	30 (88.2%)	70 (90.9%)		40 (90.9%)	30 (88.2%)	
Irregular	3 (17.6%)	0	4 (11.8%)	7 (9.1%)		3 (6.8%)	4 (11.8%)	
Margin					0.335			0.162
Well‐circumscribed	11 (73.3%)	18 (66.7%)	29 (82.9%)	58 (75.3%)		29 (69.0%)	29 (82.9%)	
Rough	4 (26.7%)	9 (33.3%)	6 (27.1%)	19 (24.7%)		13 (31.1%)	6 (27.1%)	
Degeneration								
Calcification	5 (29.4%)	7 (25.9%)	2 (5.7%)	14 (17.7%)	0.043	12 (27.3%)	2 (5.7%)	0.012
Hemorrhage	8 (47.1%)	14 (51.9%)	2 (5.7%)	24 (30.4%)	0.000	22 (50.0%)	2 (5.7%)	0.000
Septa	6 (35.3%)	4 (14.8%)	0	11 (13.9%)	0.01	10 (22.7%)	0	0.002
Multiple lymph nodes	5 (29.4%)	9 (33.3%)	8 (22.9%)	22 (27.8%)	0.818	14 (31.8%)	8 (22.9%)	0.452
Regional lymph node	1 (5.9%)	0	2 (5.7%)	3 (3.8%)	0.23	1 (2.3%)	2 (5.7%)	0.367
Retroperitoneal lymph node	4 (23.5%)	7 (25.9%)	5 (14.3%)	16 (20.3%)	0.901	11 (25%)	5 (14.3%)	0.648
Lymphoglandular mesenterica	3 (176%)	5 (18.5%)	3 (8.6%)	11 (13.9%)	0.151	8 (18.2%)	3 (8.6%)	0.128
Pelvic lymphnode	1 (5.9%)	3 (11.1%)	1 (2.9%)	5 (6.3%)	0.317	4 (9.1%)	1 (2.9%)	0.179
Inguinal glands	2 (11.8%)	3 (11.1%)	4 (11.3%)	9 (11.4%)	0.866	5 (11.4%)	4 (11.3%)	0.50

Of the 35 patients assessed by abdominal ultrasonography, RS appeared as a solid or cyst mass with clear boundaries and hypoechogenicity, and 2 of these masses exhibited rich blood flow signals.

**FIGURE 1:**
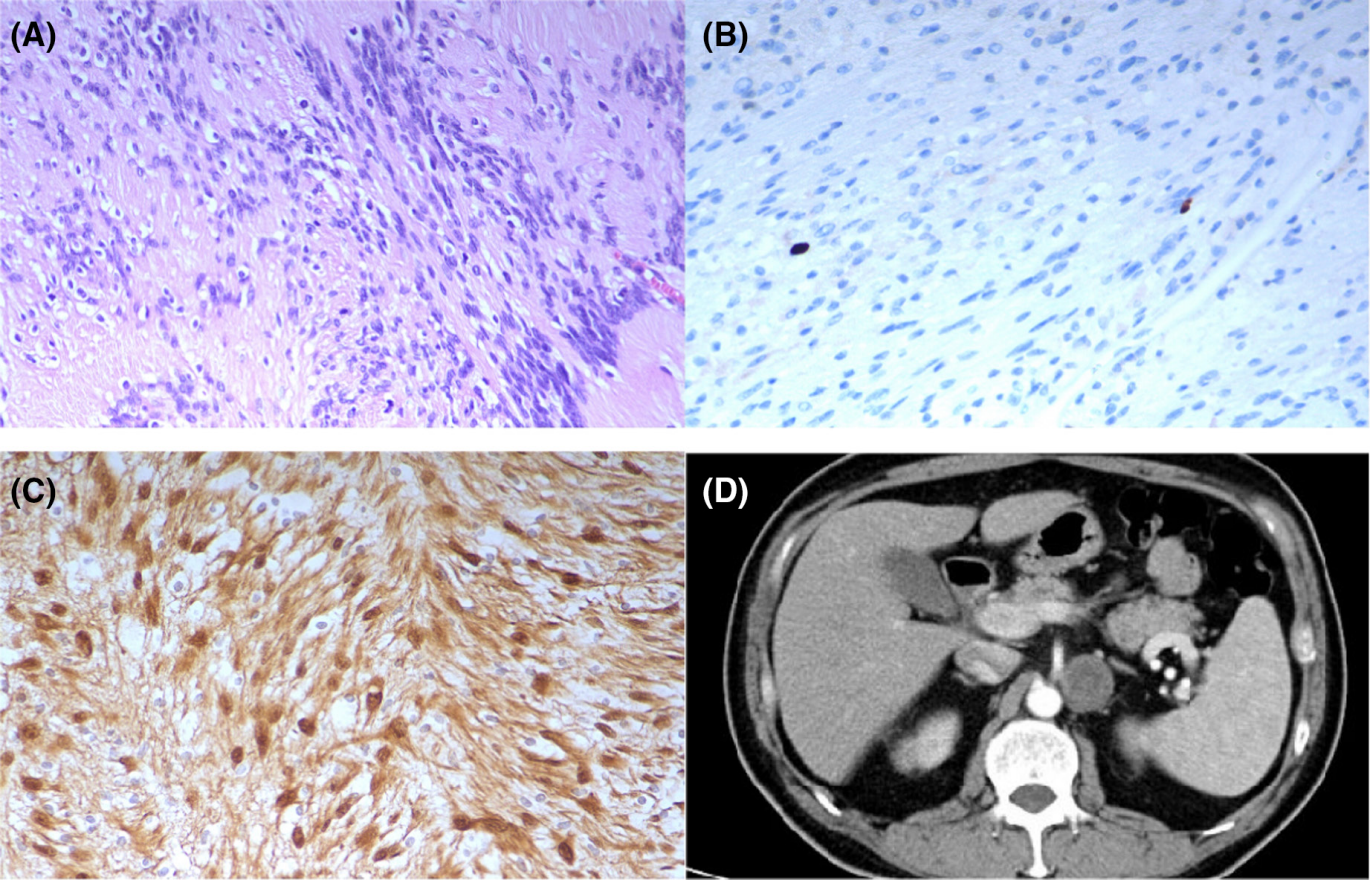
Benign retroperitoneal schwannoma of periaortic region in a 58‐year‐old man. (A) Characteristic aggregation of fibrillary, elongated cells with rare cellular atypia or mitosis figure (HE, ×200). (B) Immunohistochemical staining showed a Ki‐67 index <2%. (C) Immunohistochemical staining showed that tumor cells were strongly positive staining for spindle cells (S100, ×200). (D) Enhanced CT scan showed a rounded, well‐circumscribed solid‐cystic soft‐tissue mass on the left side of abdominal aorta. The cystic region did not enhance, while the solid portion exhibited mild enhancement.

**FIGURE 2:**
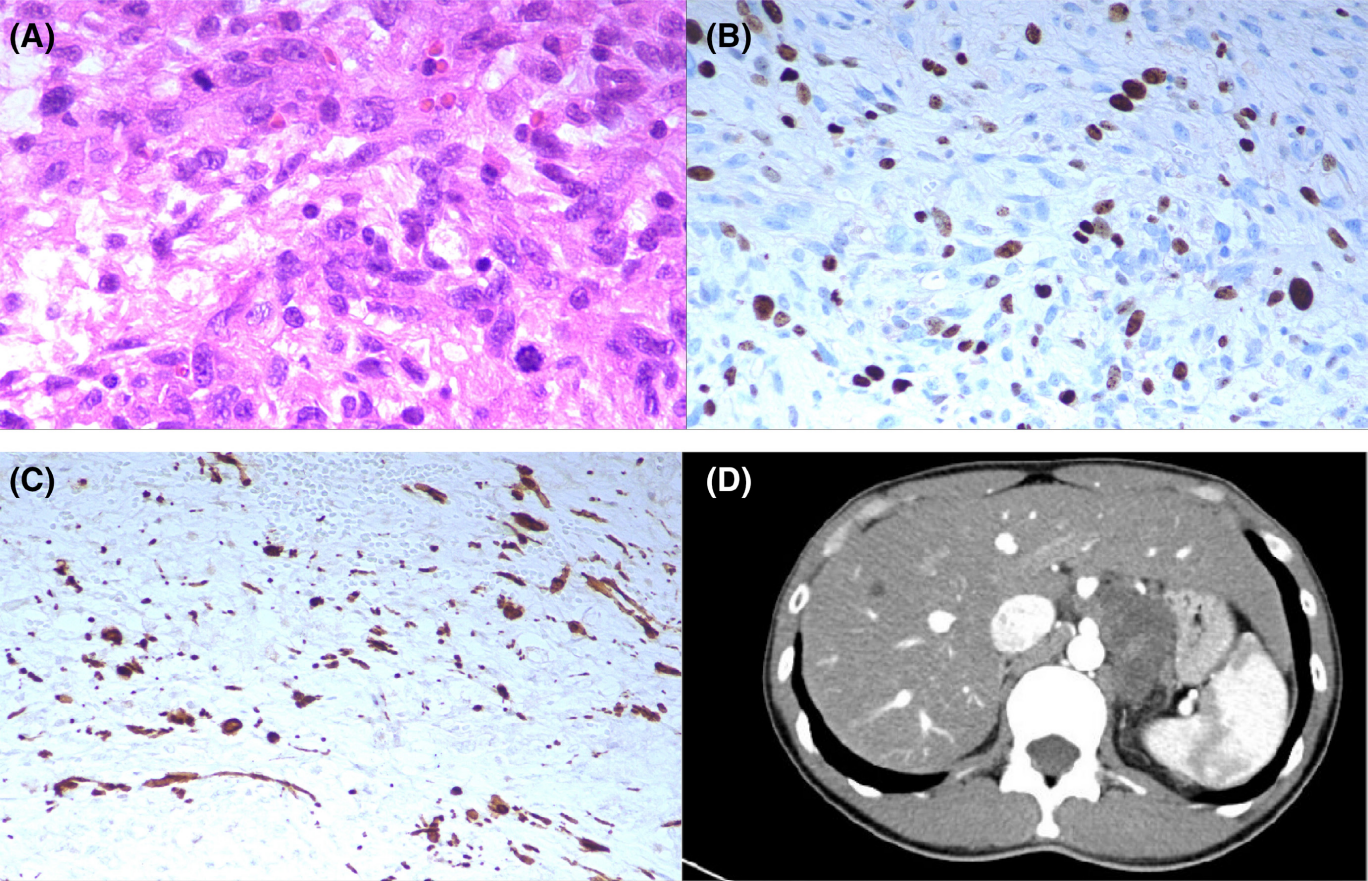
Malignant retroperitoneal schwannoma of periaortic region in a 27‐year‐old man (patient 1). (A) Atypical large nuclei and mitosis (HE, ×400). (B) Immunohistochemical staining showed a Ki‐67 index >30% (Ki‐67, ×200). (C) Immunohistochemical staining showed that tumor cells were positive staining for spindle cells (S100, ×200). (D) Enhanced CT scan showed an irregular‐shaped solid‐cystic soft‐tissue mass with rough margin between abdominal aorta and lesser gastric curvature. The cystic region did not enhance, while the solid portion exhibited mild enhancement.

Seventy‐six patients underwent CT, which revealed a rounded, well‐circumscribed or irregular, smooth or rough, hypodense soft‐tissue mass (Figures 1 and 2). The cystic region did not enhance, while the solid portion of the tumor exhibited mild‐to‐moderate enhancement on the enhanced CT scan.

Twenty‐five patients underwent MRI, which revealed an isointense signal on T1‐weighted imaging (T1WI) and an isointense or hyperintense signal on T2‐weighted imaging (T2WI). Eight patients underwent an enhanced MRI scan, which revealed mild heterogeneous and delayed progressive enhancement. No enhancement was detected in the cystic region, whereas the walls or internal septa of cysts were enhanced in 6 patients. Diffusion‐weighted imaging (DWI) showed high signal intensity in 6 patients, revealing that the diffusion of water molecules was impeded.

All tumors were unilateral. No side predilection was observed. Mean tumor diameter was 6.23 cm (range, 2.1–20.3). Most tumors were smaller than 10 cm in diameter. Tumor diameter did not significantly differ between RSs with cystic degeneration and those without.

Tumor location was as follows: paravertebral region (*n* = 22, 27.8%), pancreatic region (*n* = 5, 6.3%), adrenal region (*n* = 12, 15.2%), adjacent to renal hilus (*n* = 19, 24.1%), periaortic region (*n* = 11, 13.9%), and pelvic cavity/iliac fossa (*n* = 10, 12.7%). Among the 22 paravertebral tumors, 6 exhibited extension into the spinal canal (27.3%).

Multiple tumors were observed in 6 patients (two in group 1 and four patients in group 2). The prevalence of multiple tumors was significantly higher in patients with cystic RSs than in those without (*p* = 0.025).

Most tumors were round or oval (*n* = 70, 90.9%); the others exhibited an irregular, multilobulated appearance (*n* = 7, 9.1%). The margin was well defined in 58 tumors (75.3%) and indistinct in 19 (24.7%). Tumors with well‐defined margins did not invade adjacent structures; those with indistinct margins were in contact with the adrenal gland, psoas major muscle, spine, ureter, intestinal wall, and various blood vessels.

In this study, 44 cases (55.7%) exhibited cyst formation, 15 cases (19.0%) showed punctate calcification related to the tumor wall, 24 cases (30.4%) had hemorrhage, and 11 cases (13.9%) had internal septa. Cystic degeneration was related to calcification (*p* = 0.012), hemorrhage (*p* = 0.000), and internal septa (*p* = 0.002). The distribution of internal septa illustrates that a mass with multiple cystic lesions can be seen in large cystic RSs. Multiple slightly enlarged lymph nodes, mainly the retroperitoneal lymph node (*n* = 16) and mesenteric lymph node (*n* = 11), were observed in 22 cases.

Surrounding organs and tissues, including the diaphragm, mesentery, duodenum, jejunum, colon, pancreas, adrenal gland, kidney, ureter, spleen, psoas muscle, and lumbar vertebrae, were compressed. Similarly, these tumors compressed the abdominal aorta, coeliac trunk, superior mesenteric artery, inferior vena cava, renal artery, splenic vein, and iliac vessel, without definite invasion. RSs are hypovascular tumors, and definite supply vessels, a fraction of the minor branches of the adjacent artery, which provides perfusion routes, were observed in only 12 masses.

### 3.4 Treatment and prognosis

Table 3 summarizes patient treatment. All patients underwent pathologically confirmed complete resection (R0). Laparoscopic surgery was performed in 38 patients (48.1%); the others underwent laparotomy. Extensive resection (partial adrenalectomy) was performed in 4 patients because an indistinct demarcation was in close contact with the adrenal gland, and all 4 patients had solid tumors. Nine patients underwent adhesiolysis during surgery owing to the presence of severe adhesions adjacent to the tumor; no injury to adjacent organs occurred.

**TABLE 3:** Treatments for the 79 patients with RS

Strategy	Group 1 (*n*)	Group 2 (*n*)	Group 3 (*n*)	Total (*n*)
Surgery				
Laparoscopic surgery	6	14	18	38
Laparotomy	11	13	17	41
Resection margin				
R0	17	27	35	79
R1/R2	0	0	0	0
Extensive resection (partial adrenalectomy)	0	0	4	4
Radiotherapy	0	1	0	1
Chemotherapy	0	2	0	2
Complications				
Neurologic symptoms	2	2	0	4

Seven patients experienced postoperative complications (8.9%). No deaths occurred. Four patients with paravertebral region or iliac fossa tumors reported weakness and abnormal sensation in the lower extremities after surgery. None had preoperative neurological symptoms. Incidence of neurological complications did not significantly differ according to tumor location (*p* = 0.057). One patient initially complained of dull abdominal pain combined with nausea and vomiting, one complained of waist pain combined with nausea and vomiting, one complained of waist pain, and one presented with an asymptomatic abdominal mass. All patients initially complained of symptoms other than nerve compression symptoms. On the postoperative examination, all 4 patients had bilateral lower‐extremity muscle strength graded at 3/3+. At discharge, neurologic status had slightly improved after treatment with methycobal. One patient was lost to follow‐up. The remaining three experienced full recovery of muscle strength at 3, 6, and 12 months, respectively. There was no evidence of other operation‐correlated complications during the follow‐up. The remaining postoperative complications, including glottic edema, venous thrombosis, and lymphatic fistula, were completely improved after treatment. All patients' symptoms were greatly improved, and any additional operation‐correlated complications were not observed during the follow‐up.

**FIGURE 3:**
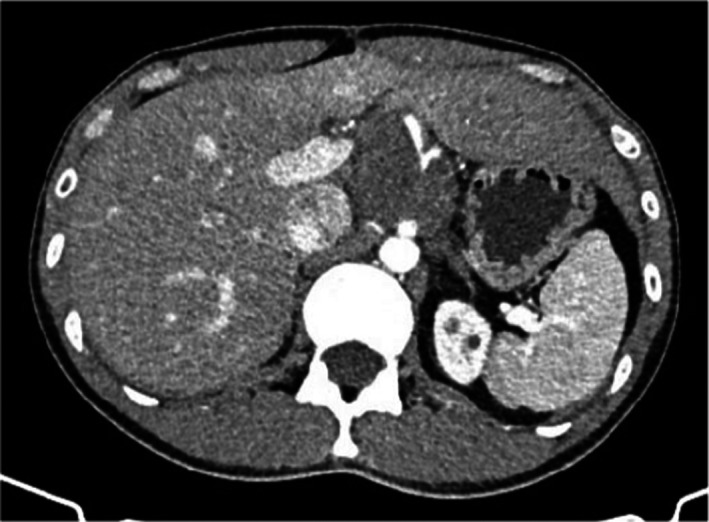
Patient 1: a recurrent tumor diagnosed 3 months after surgery surrounded the superior mesenteric artery and abdominal aorta. Enhanced CT scan showed a round solid‐cystic soft‐tissue mass with irregular enhancement surrounding the coeliac trunk and superior mesenteric artery.

**FIGURE 4:**
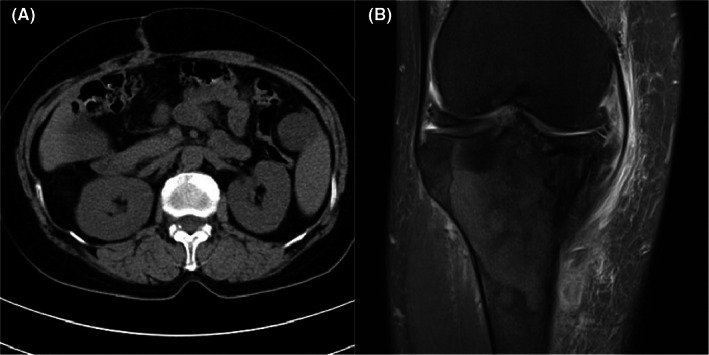
Recurrences in patient 3: (A) CT scan showed a recurrent tumor in the perisplenic region 14 months after surgery. (B) MRI showed a second recurrence in right tibial metaphysis and upside of right tibial shaft 26 months after surgery.

All three patients diagnosed with a malignant RS underwent radiologic surveillance and experienced tumor recurrence (mean follow‐up, 33 months). One recurrent tumor diagnosed 3 months after surgery surrounded the superior mesenteric artery and abdominal aorta (Figure 3). This patient complained of moderate dull abdominal pain; however, he refused adjunctive therapy and elected to continue with serial imaging. Another patient experienced recurrence near the surgical site, which was resected in another hospital 8 months after the first operation. Six months later, a second retroperitoneal recurrence appeared and was treated with immunotherapy (PD‐1 inhibitor) and targeted therapy. This patient is currently being treated and followed. The third patient underwent regular chemotherapy after a perisplenic recurrence was diagnosed 14 months after the initial operation. Twelve months later, he complained of lower limb pain and a second recurrence in the upside of right tibial shaft was diagnosed (Figure 4). This patient died with a multifocal tumor 5 months after the second recurrence.

None of the 76 patients with benign RS received radiotherapy or chemotherapy. In the most recent follow‐up (mean 45 months), no evidence of recurrence was observed in 76 patients with benign RS according to the abdominal CT scan.

### 3.5 Intraoperative and pathologic findings

Most tumors were well circumscribed with a complete capsule and did not invade adjacent structures. Tumors in the cystic degeneration group appeared red–brown or yellow–white, had a cut surface, and an associated cyst. Those in the solid group appeared yellow–white and had a fragile or firm texture with soft contents. Gross examination of the tumors showed areas of hyalinization, thrombosis, and septa. All tumors were microscopically characterized by proliferating fusiform cells.

Immunohistochemical findings are summarized in Table 4. All tumors were positive for vimentin and negative for EMA. S‐100 and SOX10 staining was positive in 98.7% and 85.7% of tumors, respectively. CD34 and GFAP staining was positive in 73.9% and 71.43%, respectively. SMA staining was negative in 79.7% and CD117 was negative in 94.1%. The histopathology of benign RSs was identical to that of other benign schwannomas.

**TABLE 4:** Immunohistochemical findings of RS patients

	Group 1 (*n*)	Group 2 (*n*)	Group 3 (*n*)	Total (*n*)	*p* value
S‐100 (+)	17	26	35	78 (98.7%)	
S‐100 (−)	0	1	0	1 (1.3%)	
SOX10 (+)	2	3	7	12 (85.7%)	
SOX10 (−)	0	1	1	2 (14.3%)	
SMA (+)	3	5	6	14 (20.3%)	
SMA (−)	11	18	26	55 (79.7%)	
EMA (−)	2	4	4	10 (100%)	
CD34 (+)	5	12	17	34 (73.9%)	
CD34 (−)	4	2	6	12 (26.1%)	
Ki‐67 < 20%	14	20	29	63 (94.0%)	0.709
Ki‐67 ≥ 20%	1	2	1	4 (6.0%)	0.709
Ki‐67	6.0 (S.D. = 7.91)	5.50 (S.D. = 8.65)	2.96 (S.D. = 1.75)	4.45 (S.D. = 6.347)	0.244
Benign	16 (93.8%)	25 (92%)	35 (100%)	76 (96.2%)	0.28
Malignant	1 (6.2%)	2 (8.0%)	0	3 (3.8%)	0.28
Vimentin (+)	4	9	10	23 (100%)	
Desmin (+)	1	2	0	3 (6.1%)	
Desmin (−)	11	14	21	46 (93.9%)	
AE1/AE3 (+)	0	1	0	1 (6.2%)	
AE1/AE3 (−)	3	6	6	15 (93.8%)	
PGP9.5 (+)	3	6	4	13 (92.9%)	
PGP9.5 (−)	0	1	0	1 (7.1%)	
DOG‐1 (+)	1	1	0	2 (9.5%)	
DOG‐1 (−)	3	6	10	19 (90.5%)	
GFAP (+)	1	2	2	5 (71.43%)	
GFAP (−)	1	0	1	2 (28.57%)	
CD117 (+)	2	0	0	2 (5.9%)	
CD117 (−)	5	10	17	32 (94.1%)	
SDHB (+)	1	1	4	6 (86.7%)	
SDHB (−)	0	1	0	1 (13.3%)	

Seventy‐six patients (94.9%) had a Ki‐67 proliferation index less than 20% (Figure 1), and 3 patients (5.1%) had a Ki‐67 proliferation index greater than 20% (Figure 2). The cystic degeneration group seemed to have a higher rate of malignant change than the solid group (6.8% vs 0), although the difference was not significant (*p* = 0.115).

Differences between benign and malignant RSs are summarized in Table 5. Forty‐four tumors with cystic degeneration were typical schwannomas with cyst formation caused by degenerative changes. Among these, 41 were benign cystic schwannomas and three were malignant cystic RSs.

**TABLE 5:** Differences between benign and malignant RSs

	Malignant (*n*)	Benign (*n*)	*p* value
Number of patients	3	76	
Age	45.67 (S.D. = 16.44)	47.22 (S.D. = 14.86)	0.86
Mean diameter (cm)	9.23 (S.D. = 6.12)	6.10 (S.D. = 3.42)	0.135
Margin			0.578
Well‐defined	2	56	
Rough	1	18	
Morphology			0.748
Regular	3	63	
Irregular	0	7	
Number of foci			0.086
Multiple foci	1	5	
Single focus	2	71	
Multiple lymph nodes	3	19	0.034
Without multiple lymph nodes	0	31	
Multiple retroperitoneal lymph nodes	3	13	0.069
Without multiple retroperitoneal lymph nodes	0	16	
Immunohistochemical examination			
S100 (+)	2	76	
S100 (−)	1	0	
Sox10 (+)	0	12	
Sox10 (−)	1	1	
Sma (−)	2	53	
Sma (+)	1	13	

The mean size of the benign RSs (6.1 cm, S.D. = 3.4 cm) was not significantly smaller than that of the malignant RSs (9.2 cm, S.D. = 6.1 cm; *p* = 0.13). Contiguity with adjacent structures was identified in 18 (24.3%) of the 74 benign RSs and in 1 of the 3 (33.3%) malignant RSs (*p* = 0.578). All three malignant cases were round or ovoid, and only one case was noted as multiple lesions. Malignant RSs tended to correlate with multiple lymph nodes (*p* = 0.034), especially retroperitoneal lymph nodes, but not to multiple foci (*p* = 0.086). Therefore, pathology is difficult to distinguish by imaging, even when combined with clinical imaging.

Staining for S‐100 protein was diffuse and strongly positive in all tumors except for one malignant schwannoma. SOX10 staining was negative in two tumors, one of which stained positive for S‐100 and SMA; the other was a malignant RS. S‐100 and SOX staining may be negative in malignant schwannomas.

## 4 DISCUSSION

RSs are relatively rare indolent tumors. The prevalence of malignancy was 3.8% in our study; therefore, the possibility of malignancy should be considered. An accurate preoperative diagnosis of RS assists in selecting treatment and avoiding unnecessary extensive resection of structures adjacent to the tumor. However, distinguishing malignant and benign RS is extremely difficult because their clinical symptoms, physical examination findings, and imaging findings are similar.^17^, ^18^

In our study, prevalence of RS was higher in young‐ and middle‐aged patients than older patients, which is consistent with other studies.^1^, ^3^ Although previous studies have reported equal morbidity between men and women, in the solid group of our study, morbidity was slightly higher in women. The most common tumor locations were paravertebral region, adrenal region, and juxtarenal region. As reported in other studies,^1^ these abdominal masses were usually discovered without symptoms by various imaging modalities as an incidental finding during a routine health examination.

### 4.1 Clinical manifestations

RS manifestations are nonspecific because of the pliability of the retroperitoneal cavity and are related to tumor effects on surrounding organs and structures. Furthermore, they are frequently atypical when present. Most RS patients usually do not experience overt symptoms because of their slow growth rate and low potential for malignancy. Clinical symptoms do not usually appear until the tumor grows to a substantial size and compresses or displaces surrounding organs.^2^ These symptoms may be nonspecific and diverse and depend on tumor size and location.

In many cases, an indolent RS develops and grows slowly over time, eventually causing abdominal symptoms such as slowly progressive abdominal pain, distension, waist pain, or palpable abdominal mass. Lower limb numbness and pain may result from nerve compression. Gastrointestinal obstruction symptoms such as nausea, vomiting, and constipation are rare.

Symptoms vary according to tumor location and size. Tumors located in the pelvic cavity were frequently discovered by the patient as a palpable abdominal mass (*n* = 3, 42.9%). Pancreatic region tumors frequently caused abdominal distension (*n* = 3, 60.0%) owing to compression of the intestinal tract. Mean tumor diameter was significantly larger in patients with a palpable tumor than other tumors (9.03 vs. 5.99 cm; *p* = 0.039).

Physical examination findings were also associated with tumor location and size. Patients who experienced tenderness or percussion pain had deep lesions. Those with percussion pain had masses that were frequently located in the paravertebral region (*p* = 0.018). Tenderness was associated with tumors located in the pelvic cavity or iliac fossa (*p* = 0.051). Most RSs were not palpable, possibly because of their small size and/or deep location. Palpable ones were frequently located in the paravertebral region, pelvic cavity, and iliac fossa (*p* = 0.001). Mean tumor diameter was significantly larger in patients with a palpable abdominal mass than those without (9.39 vs. 5.64 cm; *p* = 0.001).

Misdiagnosis of RS, particularly cystic ones, is common because of their nonspecific clinical and radiological features. These tumors may mimic an adrenal malignant adenoma,^19^ pancreatic tumor,^20^ mesenteric tumor,^21^ pheochromocytoma, paraganglioma, liposarcoma,^22^ malignant fibrous histiocytoma,^23^ ovarian carcinoma,^24^ leiomyoma, and so on.

### 4.2 Imaging manifestations

Although preoperative imaging is performed to aid diagnosis of RS, imaging studies are frequently inconclusive. Accurate preoperative diagnosis of RS is extremely difficult based on radiologic features alone.^2^, ^17^

On CT, cystic degeneration group tumors typically appeared as well‐defined, unilateral, regular, heterogeneous masses with areas of central cystic necrosis,^17^ marginal punctate calcification, or hemorrhage. Enhancement was either mildly heterogeneous or along the tumor rim. Most schwannomas appeared heterogeneous with non‐enhancing cystic or necrotic areas. This typical appearance is key when formulating a differential diagnosis. Solid group tumors were well‐circumscribed hypodense masses with annular or delayed heterogeneous enhancement. Unfortunately, CT values were not standardized in our study, so subgroup comparisons were not performed.

MRI can provide a more accurate preoperative diagnosis than CT and ultrasonography, as it better demonstrates tumor origin, extent, and internal composition.^16^ In most neurogenic tumors, signal intensity is low in the center and high in the periphery.^25^, ^26^ In our study, most RSs exhibited low or intermediate signal intensity on T1‐weighted imaging and markedly increased signal intensity on T2‐weighted imaging. However, ones with intratumoral hemorrhage showed mixed signal intensity on both sequences. Small tumors tended to enhance uniformly, while large ones enhanced heterogeneously.

RS morphology varied according to location and size. Large size was significantly associated with deep location, irregular margin, and multiple foci.

RSs are relatively larger than other schwannomas. Mean tumor diameter was 6.23 cm in our series. As a result, RSs usually exhibit spontaneous degenerative changes. Cystic degeneration presents as a tumor with single or multiple cysts. The mechanism underlying intratumoral cyst formation is not clear but may be related to tumor size: blood supply may be compromised in portions of large tumors, which results in intratumoral liquefactive necrosis, hemorrhage, and calcification.

A previous study documented that a well‐defined margin and a regular shape may suggest benignity and that contiguity between RS and adjacent neural structures may indicate malignancy.^19^ Paradoxically, this study of malignant RS may contradict this hypothesis. These features do not allow the exclusion of malignancy, providing an overly optimistic view of survival for patients with such features. Such biological behavior may illustrate that rapid growth of a malignant RS leads to the formation of a capsule and well‐defined margin, but it does not absolutely indicate that the tumor is noninvasive and benign. Radiological manifestations reflect the tumor's intrinsic behavior/characteristics and thus the heterogeneous behaviors of RSs. Cyst formation accompanying malignant RS may indicate an expansive or rapid growth pattern rather than aggressive behavior or an infiltrative growth pattern.^18^ This may also indicate that an early operation can be considered in some patients.

Cyst formation is significantly associated with calcification, hemorrhage, and presence of internal septa.^27^ Cyst formation, calcification, and hemorrhage are rare in other primary retroperitoneal tumors; therefore, RS should be suspected in a unilateral well‐defined heterogeneous mass with heterogeneous or delayed enhancement. Imaging findings combined with clinical history and laboratory findings can aid in making an accurate diagnosis. Malignant RS should be considered when a mass has multiple lymph nodes enlargement (*p* = 0.034), especially retroperitoneal lymph nodes.

In addition, neurofibromatosis and schwannomatosis should be considered for patients with a proven schwannoma. Brain and internal auditory meati MRI can aid in ruling out bilateral vestibular schwannomas (NF2). Patients with schwannomatosis develop tumors harboring independent somatic pathogenic variants in the *NF*_2_ gene which are not present in their constitutional DNA. Germline pathogenic variant in *SMARCB*_1_ or *LZTR*_1_ strongly suggest the diagnosis of schwannomatosis for patients with a proven schwannoma.^28^

### 4.3 Intraoperative and pathologic findings

The final diagnosis of RS is based on histopathology and immunohistochemistry.^17^ which remain the gold standard.^29^ Grossly, RSs appeared predominantly as white‐yellowish soft masses with a cut surface and with multiple focal hemorrhages and necrosis, consistent with a previous report.^1^ The diagnosis of RS was confirmed by characteristic positive immunohistochemical staining for S‐100 protein, Vimentin, and SOX10. S‐100 and Vimentin are markers of neuroepithelial differentiation. Immunohistochemical staining results of other benign schwannomas showed positivity for S‐100,^26^, ^29^, ^30^ SOX10, Vimentin,^31^ and GFAP, and negativity for SMA, EMA, CD34, and CD117. The immunohistochemistry findings in this study were mostly consistent with those from other studies (except for CD34, a biomarker of the vascular endothelium).

RS with a low Ki‐67 proliferation index may indicate a low possibility of recurrence. Ki‐67 ≥ 20% is highly predictive of MPNST, with 87% sensitivity and 96% specificity.^32^ To monitor recurrence, special attention should be paid to benign RSs with a focal Ki‐67 of 20%.

### 4.4 Treatment and prognosis

Surgical resection of RS is both diagnostic and therapeutic.^16^ The prognosis of benign RS is favorable following complete excision.^17^, ^32^ The ultimate treatment outcome depends largely on the pathological type, location, and adjacent anatomic structures.

When performing resection, the structures surrounding the tumor should be protected.^17^ In addition, preoperative^17^ or intraoperative^2^ arterial embolization can reduce the risk of severe bleeding and assist with removal. Although treatment of malignant RS has not been standardized, the first line of treatment is radical resection.^2^, ^16^ Local recurrence is the most frequent complication of MPNST resection and is attributable to positive resection margins and a high histological grade.^2^ Incidence of local recurrence is considerably higher with marginal excision than wide‐margin resection (72% vs. 11.7%).^33^ Although radical resection is not necessary for benign RSs, malignant ones require complete excision with negative microscopic margins,^34^ as they are resistant to all adjuvant, neoadjuvant, and targeted therapies.^11^, ^34^, ^35^ In patients with schwannomatosis, surgical intervention should be offered to those with painful schwannomas if resection can be performed without causing a neurological deficit. However, repeated surgery at the same site should be avoided as effectiveness of pain control diminishes with subsequent operations. Multiple operations may even result in worsening of the pain syndrome.^28^

Prognosis of benign RS is usually favorable. In our study, no recurrence of a benign tumor was observed after complete resection. Subtotal resection of benign RS may also prevent progression or local recurrence.^36^ In a natural history study of RS in 22 asymptomatic patients with mean follow‐up of 48 months, Ogose et al. reported a 1.9 mm/year average accumulative tumor growth rate; all patients remained asymptomatic at last follow‐up.^37^ Thus “wait & see” approach is reasonable for patients with asymptomatic RS. Malignant transformation was rarely observed in schwannomas while development into malignant peripheral nerve sheath tumors or angiosarcomas has been reported. In two series comprising 26 schwannoma patients who experienced malignant transformation, only one harbored an RS.^38^, ^39^

Neurologic complications tended to correlate with location (*p* = 0.057) and initial symptoms (*p* = 0.006). Three patients with masses in the paravertebral region and 1 patient with a mass in the fossa iliaca reported abnormal sensation and weakness in the lower extremities after surgery. Two patients with neurological complications initially complained of nausea and vomiting. One patient developed neurological function deficits because the operation relieved nerve compression. Symptomatic treatment (methycobal) and exercise therapy can help improve neurological function deficits.

Three patients with malignant RS experienced asymptomatic recurrence with rapid progression. Rapid progression indicates the malignant behavior of RS. Most RSs are benign, but whether a benign RS undergoes malignant changes with mutations is unclear.^40^

## 5 CONCLUSION

Accurate preoperative diagnosis of RS is usually not possible owing to its rarity and lack of distinct clinical and radiographic features. RS should be considered in the differential diagnosis for unilateral well‐circumscribed heterogeneous retroperitoneal masses with heterogeneous or delayed enhancement. Cystic degeneration was not associated with tumor size, Ki‐67, or malignancy; however, it was significantly associated with multiple foci, calcification, and hemorrhage. Indistinct margins and irregular shape do not necessarily indicate cystic degeneration or malignancy. Similarly, the presence of a capsule and well‐circumscribed margins do not always indicate benign behavior. Cystic change can occur regardless of tumor shape or margins. Malignant RS should be considered in tumors with multiple lymph nodes enlargement. Early surgical treatment should be performed when malignancy is suspected. Tumor margins, morphology, and size are not associated with malignancy. Resection of tumors adjacent to nerves may result in neurologic complications. Pathological tumor type, tumor location, and adjacent anatomic structures are associated with outcome.

## AUTHOR CONTRIBUTIONS

**Jianchun Xiao:** Conceptualization (equal); data curation (equal); formal analysis (equal); investigation (equal); methodology (equal); project administration (equal); writing – review and editing (equal). **Lizhu Cai:** Conceptualization (equal); data curation (equal); formal analysis (equal); investigation (equal); methodology (equal); project administration (equal); writing – original draft (equal). **Jun Pu:** Conceptualization (equal); data curation (equal); formal analysis (equal); investigation (equal); methodology (equal); project administration (equal); writing – review and editing (equal). **Wei Liu:** Resources (equal). **Xiaodong He:** Conceptualization (equal).

## FUNDING INFORMATION

This work was supported by the National Key Research and Development Program of China (2020YFF0305104).

## CONFLICT OF INTEREST

The authors have declared no conflicts of interest.

## ETHICS APPROVAL AND CONSENT TO PARTICIPATE

Approval was obtained from the ethics committee of Peking Union Medical College Hospital (Identifier: S‐K 1459), and the requirement for patient consent was waived for this retrospective study.

## CONSENT FOR PUBLICATION

Not applicable.

## Data Availability

The datasets used and analyzed during the study are available from the corresponding author Dr. Xiaodong He (hexiaodongbj@126.com) upon reasonable request.
